# Sick and Disabled Seamen

**Published:** 1847-04

**Authors:** 


*Sick and Disabled Seamen.*—One of the late Congressional documents comprises a letter from Dr. Ruschenberger, Surgeon to the U. S. Naval Hospital in New York, to a member of Congress, relative to the expenses of the Marine and Naval Hospitals. From it we make the following quotations respecting these important institutions.

"By an act approved July 16th, 1798, a tax of twenty cents monthly was levied on the pay or wages of all seafaring people; and, in consideration of the payment of this tax, collectors of ports are directed in the same act to 'provide for the temporary relief and maintainance of the sick or disabled seamen in hospitals.' Under this law all seafaring people who pay this tax, or 'hospital money,' are entitled to the 'relief' specified or intended.

"The rate of the tax thus levied is very heavy, as may be seen by comparing it with the amount of seamen's resources. For example, in the navy the total pay and emolument of a seaman are, annually, $144, and rations $73; equal to $217. From this sum he pays yearly $2 40, or more than one per cent, (1.10) on his total income. This is not an income tax, nor a property tax, but a tax upon the liability to misfortune of a class whose pursuit is eminently perilous to health and life.

"An act approved March 2d, 1799, levied the same amount of tax (to be applied in the same way) on persons serving in the navy. Officers, seamen and marines under this act were entitled to the same advantage as sailors in the merchant service. The fund resulting under the operation of these two acts constituted the '*marine hospital fund.*'

"By an act approved February 26th, 1811, the moneys, or tax collected from persons serving in the navy, were separated from the marine hospital fund, and constituted a fund under the title of 'navy hospital fund.' As no person in the navy had derived any advantage from the marine hospital fund between the years 1799 and 1811; and as it was fairly shown that the navy had paid at least $50,000 into it, this sum was taken from the marine hospital fund and paid over as the foundation of the navy hospital fund. By the act of 1811 the Secretary of the Navy, the Secretary of War, and the Secretary of the Treasury, were constituted commissioners of this fund; but, by an act approved July 10th, 1832, the Secretary of the Navy became the sole commissioner.

"The act of 1811 provides not only for the temporary, but also for the permanent relief of the sick and disabled officers, seamen and marines; and under its authority the naval asylum at Philadelphia was erected."

"From public documents I learn that the aggregate of hospital money collected from the merchant service for the year 1842 was $72,429 32, and the expenditure for the same year was $93,531 68; that the expense of sick and disabled seamen in the merchant service exceeded the receipts $21,102 36 for the year 1842, and for the half year ending June, 1843, the expenditure exceeded the receipts $9,129 77. Last year $25,000 were appropriated by Congress to meet the deficit of the marine hospital fund; and unless some means be devised to prevent this annual deficit, it will probably increase from year to year."

Dr. R. suggests several modes of reducing the expenditures of the marine hospital fund, and also of increasing the receipts of the navy hospital fund, but we believe no action was had by Congress on the subject.—*Boston Med. and Sur. Jour.*

